# An imaging analysis protocol to trace, quantify, and model multi-signal neuron morphology

**DOI:** 10.1016/j.xpro.2021.100567

**Published:** 2021-06-02

**Authors:** Sumit Nanda, Shatabdi Bhattacharjee, Daniel N. Cox, Giorgio A. Ascoli

**Affiliations:** 1 Center for Neural Informatics, Structures, & Plasticity and Neuroscience Program, Krasnow Institute for Advanced Study, George Mason University, Fairfax, VA 22030, USA; 2 Neuroscience Institute, Georgia State University, Atlanta, GA 30303, USA; 3 Bioengineering Department, Volgenau School of Engineering, George Mason University, Fairfax, VA 22032, USA

**Keywords:** Bioinformatics, Cell Biology, Microscopy, Neuroscience

## Abstract

We describe how to reconstruct and quantify multi-signal neuronal morphology, using the dendritic distributions of microtubules and F-actin in sensory neurons from fly larvae as examples. We then provide a detailed procedure to analyze channel-specific morphometrics from these enhanced reconstructions. To illustrate applications, we demonstrate how to run a cytoskeleton-constrained simulation of dendritic tree generation and explain its validation against experimental data. This protocol is applicable to any species, developmental stage, brain region, cell class, branching process, and signal type.

For complete details on the use and execution of this protocol, please refer to Nanda et al. (2020).

## Highlights

• A protocol to trace and produce multi-signal neural reconstructions
• Quantification of multi-signal data, with a focus on cytoskeletal morphometrics
• Simulation of virtual dendritic arbor, constrained by local cytoskeletal composition

## Graphical abstract

**Figure:**
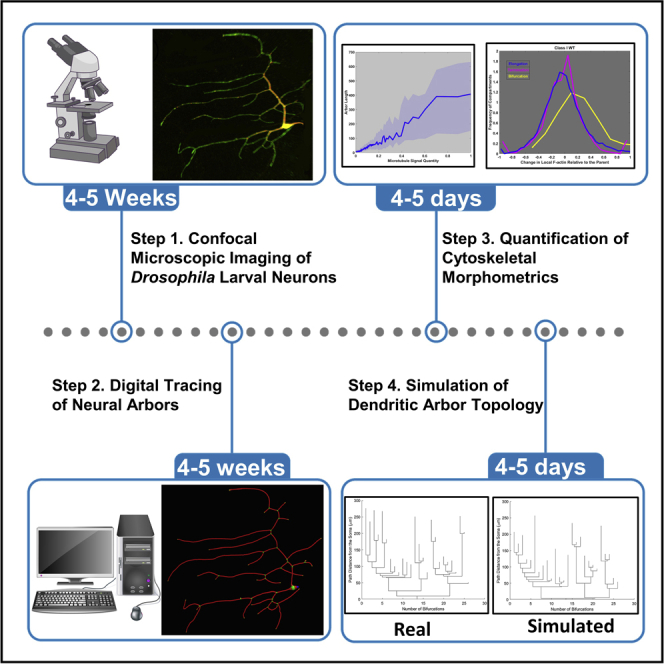

## Before you begin

Techniques for digitally reconstructing neuron morphology from light microscopy are aplenty (Acciai et al., 2016; Peng et al., 2017), and so are methods to analyze these three-dimensional structures (Ascoli et al., 2009; Polavaram et al., 2014). However, the quantification of arbor-wide subcellular signals is not as common. Here we describe a protocol to reconstruct, analyze, and simulate multi-signal neural structures, starting from confocal image stacks. While we have employed this protocol to characterize cytoskeletal distributions in invertebrate sensory neurons, this system can suit any and all imaged signals across the neural arbor. Current multi-signal characterizations are often qualitative in nature. By adapting and using the multi-signal quantification protocol described below, researchers will be able to present their observations in a measurable way. This system will, in principle, allow the subcellular characterization of any neuronal property captured through multi-channel microscopic imaging, including concentrations and spatial distributions of various molecules in cytoplasm, ion channels, cellular organelles etc.

Here, we specifically demonstrate single neuron reconstructions of larval fruit fly dendritic arborization (da) neurons followed by the quantification of microtubule and F-actin quantities. We begin from two-channel image stacks in the **.czi** file format, with the red channel representing microtubule and the green channel representing F-actin. The fly strain used for Class IV da neuron imaging is: *w1118, UAS-GMA::GFP; GAL4[477],UAS-mCherry::Jupiter*; the fly strain used for Class I da neuron imaging is: *w1118, UAS-GMA::GFP; +; GAL4[221],UAS-mCherry::*Jupiter. For overexpression of Formin3 (Form3) the following strain is used: *UAS-form3-B1* (Tanaka et al., 2004)*.*

This protocol describes the process that begins after the confocal image generation. For details on confocal imaging, see Das et al., 2017.

### Installation of analysis tools, custom codes, and example data set

1. Download the example *dataset* from Mendeley data (https://doi.org/10.17632/wpzd2wxtgn.1). Eight separate folders are included: i) Finalized Imagestacks, ii) Repair, iii) Scaling, iv) Quantify, v) Simulate, vi) TREES1.15, and vii) Vaa3D_MultiChannel_Compiled_Library, viii) StepByStepFiles.
2. Install FIJI (Schindelin et al., 2012): download the relevant zip file from imagej.net/Fiji depending on operating system and run installation executable.
3. Install Vaa3d (Peng et al., 2014): download version 3.1 from github.com/Vaa3D/release/releases/tag/v3.100 and run the installation executable.
4. To add the multi-signal reconstruction acquisition plugin (Nanda et al., 2018a) to Vaa3D, create a folder called *multi_channel_swc* under Vaa3D-3.100/bin/plugins. Copy the multi-channel compiled library (.dll for windows and .dylib for mac) from the Vaa3D_MultiChannel_Compiled_Library folder in *dataset* and place it under the Vaa3D-3.100\bin\plugins\multi_channel_swc folder.
5. Install neuTube (Feng et al., 2015): download the latest version from neutracing.com/download
6. Install MATLAB from Mathworks.com. The simulation and analyses illustrated in this protocol were tested under the MATLAB R2018a environment.
7. TREES Toolbox (Cuntz et al., 2010) and added custom functions are included as part of the *dataset.* Update the *tre_start.m* script within the repair subfolder, if required, to ensure that the address at the top of the script points to the correct TREES Toolbox subfolder under the main *Dataset.* Copy the updated *tre_start.m* script to all relevant subfolders under the Quantify folder.

## Key resources table

**Table:** 

REAGENT or RESOURCE	SOURCE	IDENTIFIER
**Software and algorithms**		

Fiji	Schindelin et al. 2012, *Nature Methods*	imagej.net/Fiji
Vaa3D 3.1 and Multi-Channel Plugin	Nanda et al. 2018a, *Scientific Data*	alleninstitute.org/what-we-do/brain-science/research/products-tools/vaa3d (download from https://github.com/Vaa3D/release/releases/tag/v3.100)
neuTube	Feng et al., 2015, *eNeuro*	www.neutracing.com
TREES toolbox	Cuntz et al. 2010, *PLOS Comput. Biol.*	www.treestoolbox.org
Matlab	mathworks.com	Version 2018a

**Experimental models: Organisms/strains**		

*Drosophila* larva, age: 4–5 days, sex: male/female *w1118, UAS-GMA::GFP; GAL4[477],UAS-mCherry::Jupiter*	Das et al., 2017, *Genetics*	Cox Lab: available by request to dcox18@gsu.edu
*Drosophila* larva, age: 4–5 days, sex: male/female *w1118, UAS-GMA::GFP; +; GAL4[221],UAS-mCherry::Jupiter*	Das et al., 2017, *Genetics*	Cox Lab: available by request to dcox18@gsu.edu
*Drosophila* larva, age: 4–5 days, sex: male/female *UAS-form3-B1*	Tanaka et al., 2004	Cox Lab: available by request to dcox18@gsu.edu

**Deposited data**		

Example data set	Nanda et al., 2020	https://doi.org/10.17632/wpzd2wxtgn.1
Neuronal reconstructions	Nanda et al., 2020	NeuroMorpho.Org*(Ascoli and Cox archives)*

## Materials and equipment

**Table:** 

Software and Algorithms	Use
FIJI	Image Preprocessing
Vaa3D 3.1 and Multi-Channel Plugin	Image Stitching, Creation of multi-signal SWC
neuTube	Tracing, Editing
TREES Toolbox	Editing, Analysis
MATLAB	Analysis and simulation Environment

Experimental Models: Organisms/Strains	Use
*Drosophila* larva (Age: 4–5 days, Sex: Male/Female) *w1118, UAS-GMA::GFP; GAL4[477],UAS-mCherry::Jupiter*	Multi-channel live imaging of F-actin and microtubule cytoskeletons in *Drosophila* Class IV da neurons
*Drosophila* larva (Age: 4–5 days, Sex: Male/Female) *w1118, UAS-GMA::GFP; +; GAL4[221],UAS-mCherry::Jupiter*	Multi-channel live imaging of F-actin and microtubule cytoskeletons in *Drosophila* Class I da neurons
*Drosophila* larva (Age: 4–5 days, Sex: Male/Female) *UAS-form3-B1*	Overexpression of wild-type full length Formin3

## Step-by-step method details

We begin by describing how to pre-process image-stacks of a Class IV WT neuron, followed by reconstruction. We then demonstrate the procedure to run neuron-group level analysis of dendritic morphology and cytoskeletal distribution for two distinct da neuron types (Class I and Class IV da neurons) as well as a genetic manipulation, namely Form3 overexpression (Form3-OE).

### Pre-processing of images (tools used: Fiji and Vaa3D)

Timing: [days to weeks]

**CRITICAL:** The raw neural images taken from the microscopes cannot be directly reconstructed in tracing tools, as a single neuron may be captured using multiple image tiles, and additional pseudo-signals are required for reliable and accurate tracing of the arbors.

**Figure 1:**
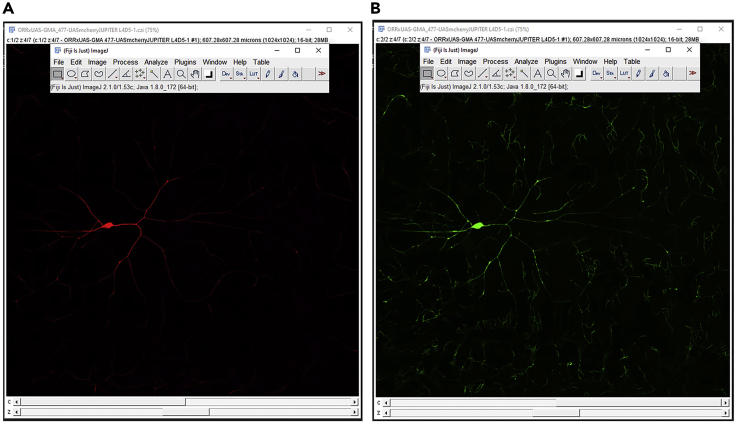
Preprocessing of image-stacks in FIJI Two-channel image with microtubule (A) and F-actin (B) signals.

1. Open the raw image file (in this specific example, the used raw image stack is in the **.czi** format) in FIJI (Schindelin et al., 2012) and save as 8 bit image in **.v3draw** format.
2. Add the red and green signals (Figure 1) using the **FIJI> process> image calculator > add signal** module to create a third pseudo channel (Figures 2B and 2C). This third channel has a higher signal-to-noise ratio and is also continuous. Hence it is used to guide the tracing process.

**Figure 2:**
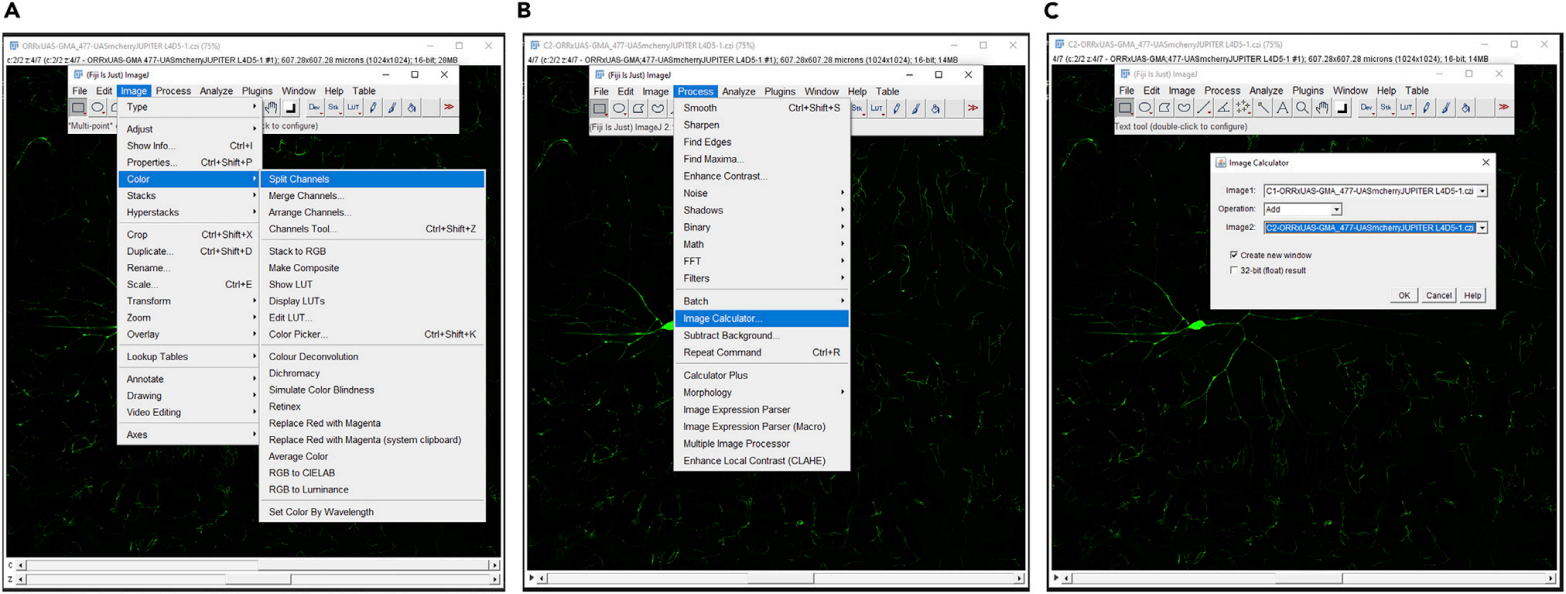
Creation of a pseudo-channel from two signals (A) The channels of an image stack can be separated using the Split channel plugin in FIJI (Image>Color>Split Channels). (B) Signals from two channels can be added using the Image calculator plugin (Process>Image Calculator). (C) Signals from two split channels can be added to create a third channel.
3. Merge the third pseudo-channel with the original two channels to create a three-channel image-stack (Figure 3).

**Figure 3:**
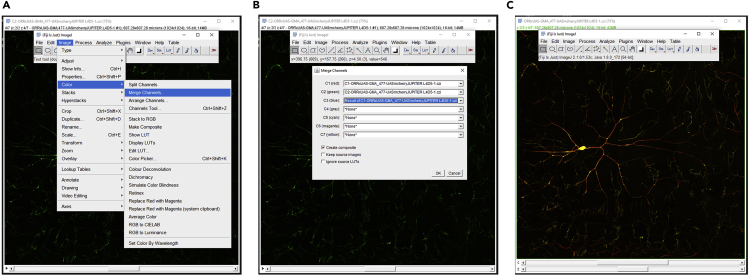
Integration of multiple signals and pseudo-channel (A) Separate channels of an Image-stack can be combined in FIJI using the Merge Channels plugin. (B) The two original channels (Figure 1A and B) can be merged together with the newly created pseudo-channel (added as the third channel). The three-channel composite image (C) can be saved.
4. Open the three channel image tiles in Vaa3D (Peng et al., 2014). Stitch together multiple image tiles in Vaa3D with the **Image-stitching >> istitch >> Pairwise Image stitching** protocol (Figure 4) using the created third channel as a reference guide.

**Figure 4:**
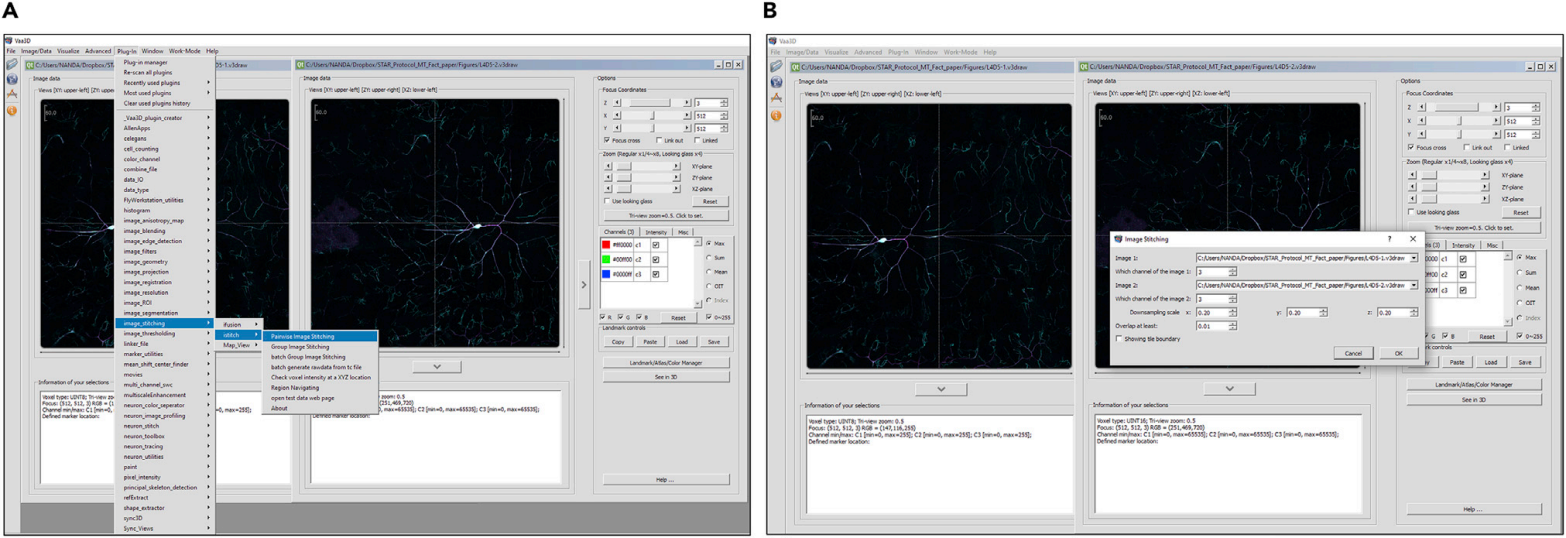
Image-stitching (A) Two image tiles of a Class IV neuron can be stitched together in Vaa3D using the Pairwise Image Stitching plug-in (Plugins>Image Stitching> iStitch > Pairwise Image Stitching). (B) In the plug-in menu, the third channel (from Figure 3C) is used to guide the stitching process.

### Semi-automatic multi-signal reconstruction of confocal neural images (tools used: neuTube and Vaa3D)

**Timing: [days to weeks]**

**CRITICAL:** Analysis of neural morphology directly from microscopic images is coarse, noise-prone, and inadequate. Digital neural reconstructions are required to precisely analyze neuron morphology.

Accurate reconstructions of the neural arbors require faithful manual input along with the semi-automated reconstruction system in neuTube. (Watch video demonstration of tracing in neuTube at youtube.com/watch?reload=9&v=QlCl_U2Zwkc&ab)

Multi-Channel plugin in Vaa3D (version 3.1) is used to create multi-signal neural reconstructions that describe morphology along with additional subcellular characteristics captured through multiple signals.

5. Open neuTube and load the three-channel stitched .v3draw image of a single Class IV da neuron.
6. Extract the third channel (**Tools >> Process >> Extract**). This will create another image of just the third channel (Figure 5). Open the image in 3D (trace mode) and use the third channel to guide the reconstruction process.

**Figure 5:**
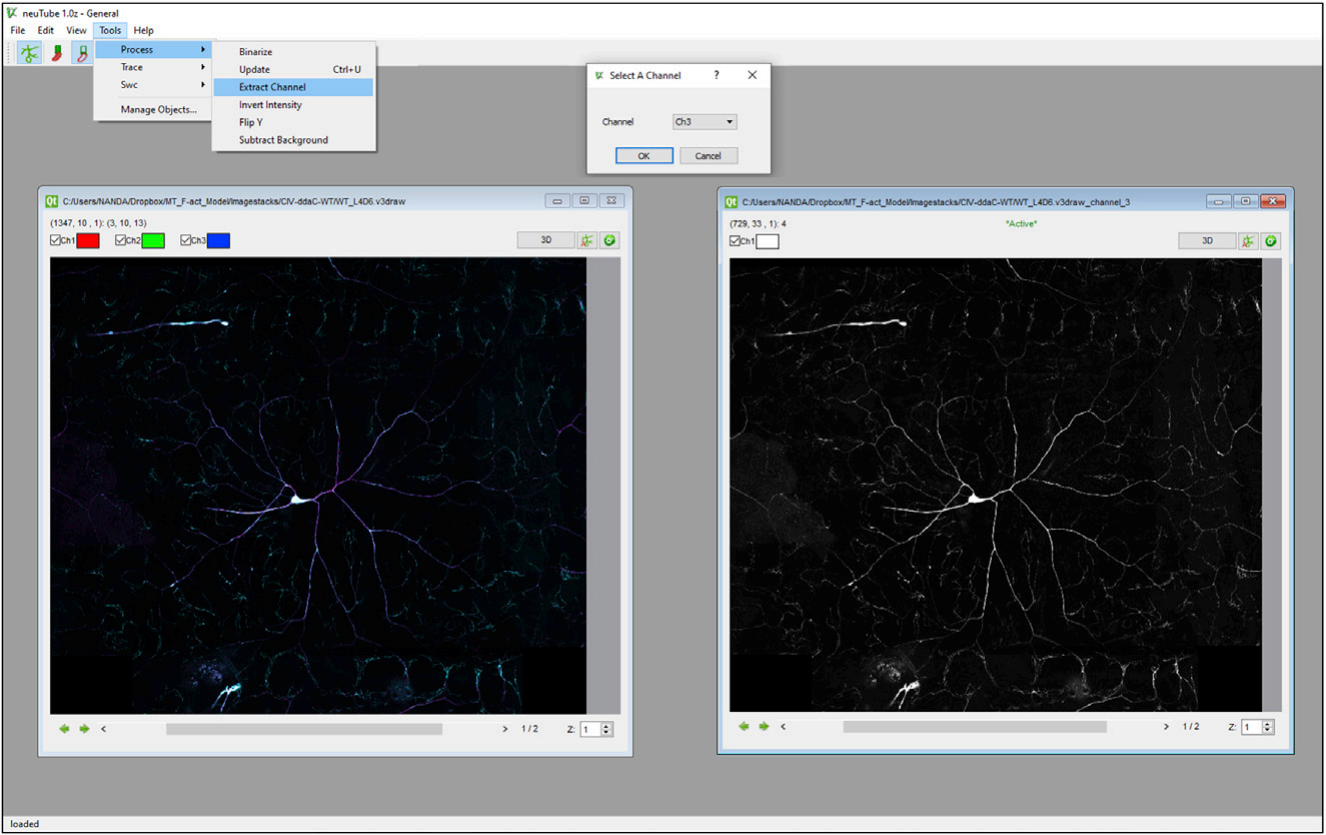
The pseudo-channel can be extracted from the three-channel image-stack in neuTube (Tools>Process>Extract Channel>select the 3rd channel) and used to guide the reconstruction process
7. Use the **Volume >> Transfer Function 1 >> adjust_signal** module to increase the intensity of the signal so that the image is clear enough to begin reconstruction. Increase the brightness and contrast by adjusting the curve (Figure 6).

**Figure 6:**
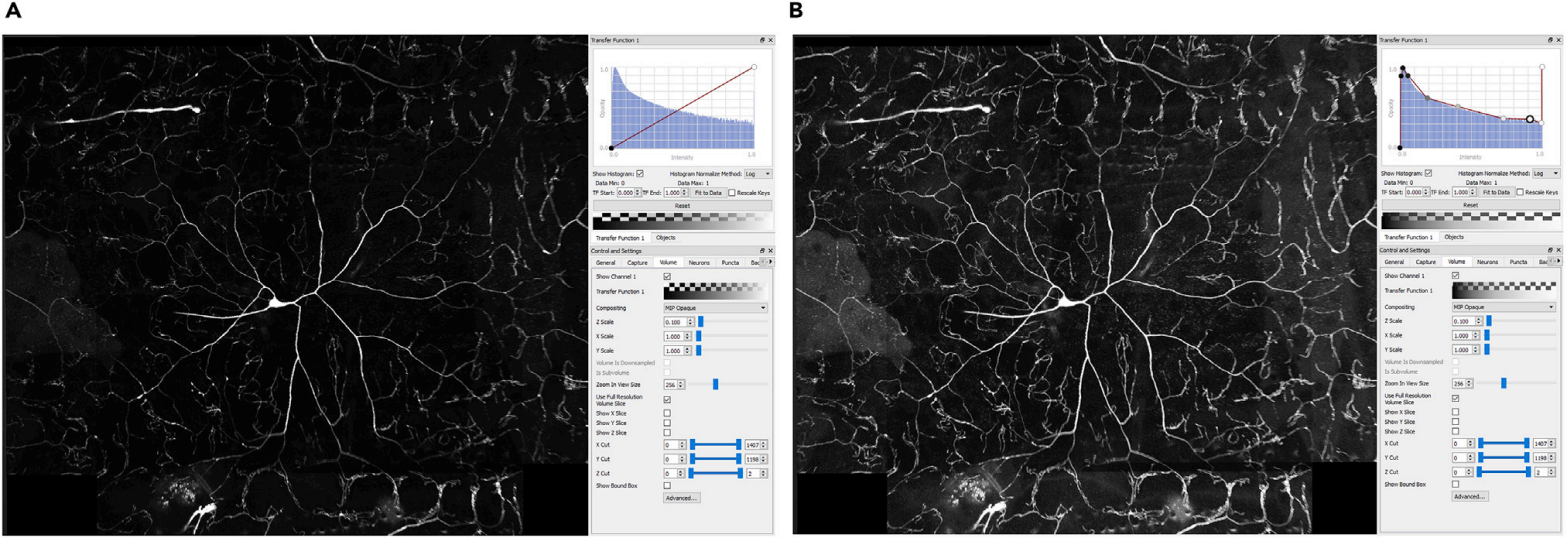
Signal intensity adjustment (A) The brightness of the third channel loaded in neuTube 3D viewer can be adjusted using the transfer function under the Volume Tab in the Control and Setting menu. (B) The red curve can be adjusted by dragging in order to make the image brighter. This process helps with the manual editing of the reconstruction.
8. Trace the brightest branches by left-clicking and selecting **trace** from the resulting menu (Figure 7A). Once the main structure is traced, trace the remaining arbors using the **extend** mode. To activate extend mode, click on an end point and press space. Press **Ctrl+S** (**Command+S** in Mac) to save the swc file. Alternatively, select the neural reconstruction in “normal mode” (under the Neurons tab in Control and Settings, see Geometry and select normal mode), right click, and then click on “save as”. Use a file name that matches the image stack name for consistency.

**Figure 7:**
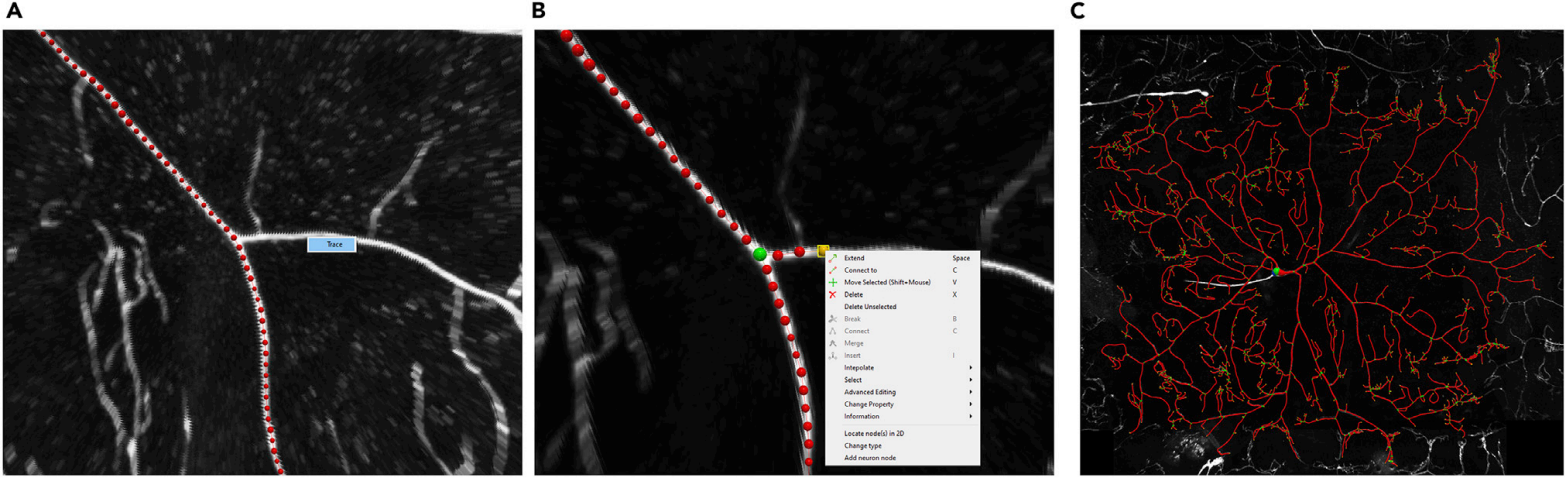
Reconstruction process in neuTube (A) The brighter signals within the image can be reliably traced automatically, by simply left-clicking and then pressing the “Trace” button. (B) For manual interventions, several operations are available in the reconstruction mode: extend, connect, move, delete etc. (C) Completed reconstruction of a Class IV WT neuron.
9. Right-click on a soma node and select as root (**change property >> set as root**). That node will then turn blue.
10. Connecting and disconnecting nodes (Figure 7):
  a. Press and hold the **‘Ctrl’** key and select two nodes, the click **‘C’** to connect.
  b. Select two or more connected nodes and press **‘B’** to break the connections.
11. Changing the size of nodes: after selecting one or more nodes, press **‘Q’** to make them smaller or **‘E’** to make them bigger. Manually adjust dendrite thickness based on the signal intensity and spread, to accurately represent local dendritic diameter.
12. Delete nodes: select nodes and hit **‘Del’** key.
13. Extend nodes: click on a terminal node and press ‘**Space’** to go into the extend mode. Left-click on image stack to extend and continue to extend by left clicking. Multiple nodes will be added to form a connection between the current selected end point and the point at which the mouse is clicked. Move individual nodes in all three dimensions by first selecting the node, then pressing **“V”,** and then dragging the node by mouse movements while pressing the **“shift”** button.
14. Where precise manual input is required, press and hold **‘ctrl’** and left-click to add single nodes per click.
15. Inserting nodes: select two or more nodes to add a node in between. Right-click and select **Insert** from the opened menu.
16. Merging adjacent nodes: select both nodes, right-click, and select **merge** from the opened menu.

**CRITICAL:** Topology of the dendritic arbor

Specific issues must be taken into consideration when tracing a neuron in neuTube. Use the sphere mode (under the Neurons tab in Control and Settings, select Sphere mode in the Geometry options; this is also the default mode) in neuTube to visualize the neural skeleton in a “ball and stick” format during the tracing process. Use the default color scheme (under the Neurons tab in Control and Settings, select Topology mode in the Color options). In this topology mode color scheme, the soma root should be the only dark blue node. If any other node turns dark blue in addition to the soma root, this indicates a fragmented subtree that must be connected to the rest of the arbor. A single neuron should have a single root node. If another node is dark blue and the soma is not, this indicates that the original root was accidentally shifted. This needs to be corrected by re-clicking on the root in the soma and setting it as the root. Branching nodes (bifurcation points) should have a light green color. Bifurcations are connected to two child nodes. If more than two child nodes of a bifurcation point exist, insert another point and create an additional bifurcation at that location (Verwer and Van Pelt, 1990). End of branch nodes (terminal tips) should have yellow color. All remaining traced nodes (continuation points) should only have red color.

**Figure 8:**
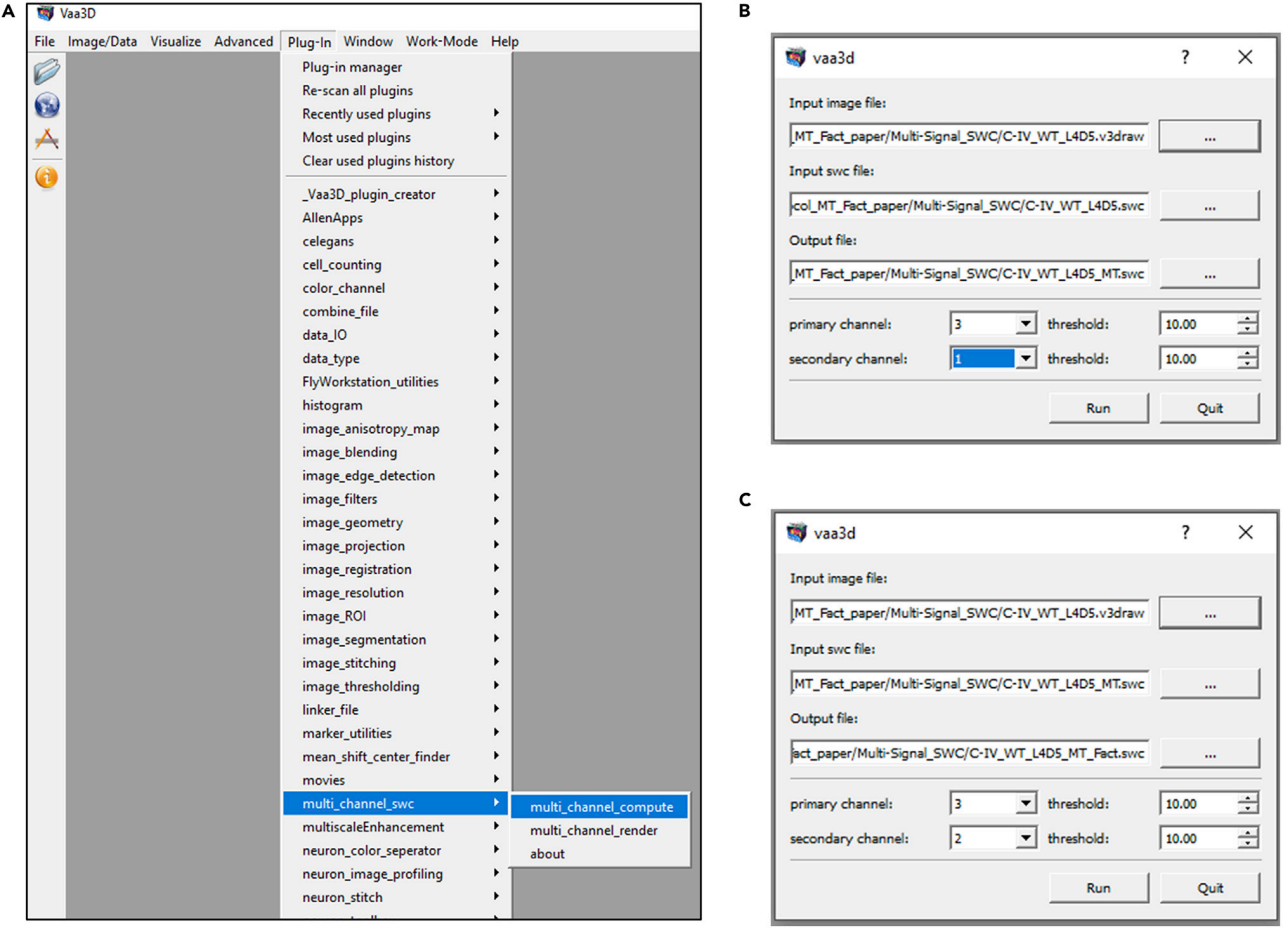
Multi-signal reconstruction acquisition plugin in Vaa3D (A) **multi_channel_swc** plugin can be accessed under the main plugin menu of Vaa3D, after the required libraries are copied (See Before You Begin section for installation details). The **multi_channel_compute** plugin interface asks for an input image file, an input swc file and an output file name. The interface also asks the user to specify the primary and the secondary channels as well as their corresponding thresholds. For two signals, the Plug-in has to be run twice. (B) In this specific scenario, the first signal is microtubule, represented in Channel 1. Therefore Channel 3 and Channel 1 are selected as Primary and Secondary channels for the first run, respectively. (C) For the second run, F-actin is measured. Therefore Channel 3 and Channel 2 are selected as Primary and Secondary channels, respectively. For all channels, a signal intensity threshold of 10 is used (in a 0 to 255 scale).
  b. This plug-in has two tools (Figure 8A):
    i. **Multichannel_Compute** to acquire multi-signal swc files.
    ii. **Multichannel_Render** to graphically represent multi-signal swc files. Click on the **Multichannel_Compute** tool to acquire multi-signal reconstructions.
  c. In the **Multichannel_Compute** interface, select the third channel (created by adding the first and the second channels) as the primary channel. Then select the first channel (in this specific case, the red channel containing the microtubule signal) as the secondary channel. Select the basic swc reconstruction file as the **Input SWC file.** Determine the optimum threshold depending on the noise level in the image stack and then apply that threshold for both the channels (Figure 8B). A threshold of 10 has been used in this example.
  d. Run the **Multichannel_Compute** plugin once (Figure 8B). This will output two multichannel files. One file details the multichannel data in the ESWC format with three additional columns for each channel, annotating (1) fraction of voxels above threshold, (2) mean intensity, and (3) standard deviation of intensity. The second file, in a back-compatible SWC format with the regular 7 columns, appends the fraction and mean of subcellular channels (in this case, the microtubule channel, since it is selected as the secondary channel) at the end of the basic neuron tree after a **#CHANNELSWC** tag.
  e. Run the **Multichannel_Compute** plugin for the second time (Figure 8C), this time using the resultant SWC file (the second output file from step 18d, containing channel information from the microtubule signal) as the input SWC file**.** The selection for the primary channel remains the same as in the first run, i.e., it consists of the third channel. Select the second channel (green F-actin signal) as the secondary channel. Choose 10 (on a 0–255 scale) as the voxel intensity threshold, signifying that any voxel below 10 will not be considered.
  f. The output file from the second run will contain signal information from both the first (microtubule) and second (F-actin) signals. Use the output swc file from this second run for the remaining quantification processes (steps 19 and beyond).

17. Postprocessing reconstructions
  a. Copy the reconstructed SWCs in the **repair** folder.
  b. Run the ***Process_tree.m*.** This script identifies and corrects topological errors in the reconstructed tree structure and repairs them; the process also sorts the neural arbors in an anterograde manner: when sorted, a parent of an **swc** compartment is closer to the soma than the compartment itself. Reconstructions can also be resampled to make all compartment lengths (adjacent node to node distance) equal. Here the neurons are resampled at 2 μm. The outputs will be generated in the **out** folder.
  c. Copy the output swc files from the **out** folder and open them back in neuTube along with the image for inspection and further edits, if required.
  d. Minor editing of the nodes manually (in neuTube) may be required since resampling (while running ***Process_tree.m***) slightly changes node locations within the arbor.
  e. Make sure all the dendritic compartments are tagged as type 3, all the axonal compartments are tagged as type 2, and all the somatic compartments are tagged as type 1. If needed, select the nodes that require updating, right click, and select “change type” from the pop-up menu. Then choose the correct types for the selected nodes.

18. Generating multi-signal ESWC files
  **CRITICAL:** Multi-signal ESWC reconstructions are generated starting from the multi-channel image data and the standard SWC reconstructions. The basic SWC file is used as input in the **Multichannel_SWC** plugin of Vaa3D along with the image stacks for each channel. Within each compartment (frustums defined by the basic SWC file), the plugin then identifies the voxels with intensities above the input threshold for the primary channel (in this case the overall morphology signal) and then checks the intensity of the same voxels for the secondary signal.
  a. Access the ***Multichannel_SWC*** plugin from the Vaa3D main plugin menu (Figure 8).
    ***Note:*** this plugin should be part of the Vaa3D main plugin menu after completing Vaa3D installation and copying the relevant libraries under the **bin/Multi_channel_swc** folder. See the **Before you Begin** section above for details on installation.

**CRITICAL:** The number of **Multichannel_Compute** runs per neuron equals the number of distinct signals that are to be measured.

19. Scale SWC
  a. Place all multi-signal SWC files (from all neuron types) in the **scaling** folder**.** This folder contains two MATLAB scripts: ***Imp_eswc.m*** and ***finalize_tree.m*.** Create a subfolder called **scaled.**
  b. Run the ***finalize_tree.****m* script. The scaling factors are hardcoded: [X, Y, Z, Radius = 0.593, 0.593, 1, 0.593] in line 17–18 of the ***finalize_tree.m*** script. Change these factors in the ***finalize_tree.m*** script, if required, based on the pixel size (X, Y, Radius) and Z-step (Z), all expressed in microns.

### Multi-signal analysis and quantification of cytoskeletal morphometrics (analysis environment: Matlab)

**Timing: [hours to days]**

**CRITICAL:** Multi-signal analysis of neuron morphology is novel. Hence, custom scripts have been created to capture subcellular characteristics simultaneously with overall morphology. In this example protocol, the final multi-signal reconstructions contained sub-structural information for both microtubule and F-actin. These could be any other set of signals, depending on the researcher’s experimental setup and the following quantifications may also require alterations depending on the scientific questions. In this specific scenario, the two signals are represented and analyzed in each compartment as cytoskeletal (microtubule and F-actin) quantity, which was calculated as a product of three parameters: average signal intensity, fraction of compartment volume occupied by the signal, and compartment thickness. The local cytoskeletal quantity, **CQ** (i.e., quantity of microtubule or F-actin signal within a compartment), is thus defined as:

CQ=F∗ASI∗W

where **F** is the fraction of the compartment volume occupied by the signal, **ASI** is average signal intensity within that fraction volume, and **W** is local dendrite width, i.e., the diameter of the compartment.

20. Quantification process
  All quantification is carried out in the MATLAB environment and extensively leverages the built-in morphological functions of TREES Toolbox.
  a. Open the Quantify folder and go into the ***Basic_Quantification*** *subfolder* in MATLAB. Copy all scaled SWC files of a given neuron type to the ***Basic_Quantification*** subfolder.
  b. Run the ***Cyto_Ana_Regular_20Col.m*** script. This script will iterate over all neurons in the folder to calculate the following 20 values for each compartment within each neuron (for detailed definitions, see Nanda et al., 2020):
    i. Topological distance from the root
    ii. Microtubule Quantity
    iii. F-actin Quantity
    iv. Diameter
    v. Branch Order
    vi. Topological event (bifurcation, elongation or termination)
    vii. Arbor length (total downstream length of dendritic arbor from the given compartment. The arbor length of the soma is that neuron’s total neurite length and the arbor length of any terminal tip is zero)
    viii. Microtubule Intensity (quantity normalized for branch thickness)
    ix. F-actin Intensity (quantity normalized for branch thickness)
    x. Relative local change in Microtubule Quantity
    xi. Relative local change in F-actin Quantity
    xii. Relative local change in Microtubule Intensity
    xiii. Relative local change in F-actin Intensity
    xiv. Path Distance
    xv. Compartment length
    xvi. Strahler Order
    xvii. Parent compartment id
    xviii. Compartment Type (axon, dendrite, soma)
    xix. Integral Microtubule
    xx. Integral F-actin
  c. A single 20 column matrix is produced as output. Update the output file name (at the bottom of the script) in the ***Cyto_Ana_Regular_20Col.m*** script according to the neuron type. For example, for Class IV Form3-OE neuron quantification, rename the output file from **C4_20Column.mat** (default name for Class IV WT) to **C4Form3OE_20Column.mat.**
  d. Run the ***TopologicalAna.m*** script to calculate the Strahler and path distance-based distribution of dendritic length, microtubule, and F-actin. This will create a file called **RealToPo.mat.**
  e. Rename the file to reflect the neuron type. For example, for Class IV wild type, rename to **RealToPo_C4WT.mat.**
  f. Remove the swc files in the **Basic_Quantification** subfolder, and then replace them with the swc files from the next neuron type. Repeat the previous five substeps (20 a, b, c, d and e) until all neuron types are complete.
  g. To create Sholl-like plots, copy the **RealToPo.mat** files from all neuron types to the **PD_Strahler_Based_Quant** folder. Current script is written to process three specific neuron types: Class IV wildtype (WT), Class I WT and Class IV Form3-OE. Edit the script to suit the specific needs for each study.
  h. Run ***norm_script.m*** to normalize cytoskeletal parameters. Cytoskeletal quantities are normalized based on average total microtubule and average total F-actin of Class IV WT neurons. All other cytoskeletal quantities are represented relative to average total Class IV cytoskeletal quantity (Microtubule or F-actin).
  i. Rename the .mat file names within the script as required.
  j. Run ***TopoAna_Graph.m*** to generate Strahler order and Path distance-based figures of length, Microtubule and F-actin. Current script is created to process three specific neuron types: Class IV WT, Class I WT and Class IV Form3-OE. Edit the script to suit the specific needs for each study.

21. Compute the Correlation between Arbor length and local topological/cytoskeletal parameters.
  **CRITICAL:** Linear (Pearson’s) correlations between local Arbor Length (total downstream arbor length) and local cytoskeletal/topological parameters are measured to determine whether microtubule (or any other parameter) is the best predictor of arbor length amongst all the measured topological and cytoskeletal parameters.
  a. Copy the C4_20Column.mat for Class IV to the ArborLength_Corr folder and run the Arbor_Len_Corr.m script to measure the correlation between arbor length and the following topological and cytoskeletal parameters:
    i. Path Distance
    ii. Branch Order
    iii. Diameter
    iv. Local Microtubule Quantity
    v. Local F-actin Quantity

22. Compute the relative local change in F-actin
  **CRITICAL:** The local change in F-actin at every compartment from its parent compartment is measured by subtracting the current quantity of F-act from that of the parent compartment. The ratio of F-act change to the parent F-act quantity yields the relative F-act change (FΔ):
  $$F\Delta =\frac{\left(\mathrm{Fc}-\mathrm{Fp}\right)}{\mathrm{Fp}}$$
  where Fp and Fc are the F-actin quantities of the parent and of the current compartment, respectively.
  a. To measure the cumulative distribution of local F-actin change separately for bifurcating, elongating, and terminating compartments, first copy the folder to the **Quantify_actin_enrichment** folder.
    ***Note:*** the **C4_20Column.mat** folder is specific for Class IV WT; use the relevant output files for Class I and Class IV Form3-OE neuron groups.
  b. Run the ***Fdelta_CurveShifts.m*** script to produce the cumulative frequency distribution curves for the three neuron types.

### Example application: Generative model of dendritic growth and comparative analysis of real and simulated neuron (simulation environment: Matlab)

**Timing: [Minutes for a single simulation run]**

A major scientific application of neuromorphological reconstruction is simulation of virtual morphology (Ascoli, 2002). These simulations are often driven by different growth rules and the reproduced morphologies are compared with their real counterparts on topology and geometry (Nanda et al., 2018b). The simulation protocol we describe here is based on the specific kind of signals measured in the above examples, namely the arbor-wide distribution of microtubule and F-actin. At each iteration, a branch within the virtual growing tree either elongates, bifurcates, or terminates depending (stochastically) on its cytoskeletal composition. For more details, see Nanda et al., 2020.

23. Simulation protocol of dendritic arbor generation
  a. Under the Simulation folder identify the subfolders corresponding to the three neuron types analyzed.
  b. To launch the model for each neuron type, run the ***Auto_M_F_Sim_MHSorted.m*** file (within the "**Simfolder**"), under every neuron type folder. The output .mat files will be generated within the "**Simfolder**". Each generated .mat file is a simulated neuron.
  c. Create artificial reconstruction (swc) files by running the ***createSwCsMultiNeuBW.m*** script within the "analysis" folder. Copy the .mat files (generated from the simulation) within the "analysis" folder and run the ***createSwCsMultiNeuBW.m*** script. Each .mat file, describing a single virtual neuron, will be converted to two swc versions with identical topology: in both cases, the column to describe diameter is repurposed to describe microtubule and F-actin quantity, respectively. Further analysis of the simulated neurons read both the files together.
  d. For each artificial neuron generated, two swc files are created: one for microtubule and the other for F-actin distribution.
24. Comparative Analysis of Simulated Neurons vs. Real Neurons
  a. Copy the simulated swc files to the **Sim_Neuron_Basic_Quantification** folder under the Quantify folder (after removing the previous swc files from the folder). Run the ***SimNeuronsProperties.m*** script to calculate for each compartment within every simulated neuron seventeen of the twenty properties calculated for the real neurons in step 20b.
  b. Run the ***TopologicalAna_Sim.m*** script to create the Strahler order and Path distance-based distributions of length, microtubule, and F-actin for the simulated neurons.
  c. To compare the topological and cytoskeletal distribution for all neuron types, move the three real and the three simulated neuron group outputs to the **Comparative_Analysis_of_real_and_Simulated_Neurons** folder under the **Quantify** folder.
  d. Run the ***Comparative_Topological_Cytoskeletal_Dist.m*** script to generate the graphs that display the simulated neuron distribution in dark thin lines and the real distributions in the background with lighter, thicker lines.

## Expected outcomes

Three neuron types are described as part of this protocol. Correlation between arbor length and local microtubule quantity is highest amongst all topological and cytoskeletal parameters for all neuron types. See Table 1 in Nanda et al., 2020 (https://doi.org/10.1016/j.isci.2020.101865) for correlations between arbor length and cytoskeletal/topological parameters. F-actin is enriched at the dendritic branch points for Class IV and Class I neurons, whereas Class IV Form3-OE neurons demonstrate lower levels of enrichment. See Figure 3 in Nanda et al., 2020 (https://doi.org/10.1016/j.isci.2020.101865), for association of F-actin enrichment with dendritic branching. Comparison of real and simulated neurons from the three neuron types used as example in this study demonstrates equivalent topological structure and cytoskeletal distribution. See Figures 5 and 6 in Nanda et al., 2020 (https://doi.org/10.1016/j.isci.2020.101865), for comparison of cytoskeletal morphometrics between real and simulated neurons.

## Limitations

Multi-signal image-stacks that contains subcellular signals are even more difficult to trace than image stacks with simple membrane signals. Automated techniques are yet to achieve that precision, hence focused manual intervention is required. The protocol is limited as it does not quantify dynamic structural and cytoskeletal changes. Future protocols will reconstruct time-varying neural structures along with cytoskeletal dynamics. For examples of recent advances in dynamic neuron analysis and modeling, see Sturner et al., 2019 and Castro et al., 2020, respectively. Future modeling protocol will be driven by time-varying cytoskeletal dynamics and will attempt to simulate both topology and geometry, including dynamic structural phenomena such as scaling and self-avoidance.

## Troubleshooting

Re-install specific tools if errors persist while using them.

### Problem 1

**Topological Error in trace** (step 16)**:**

The reconstructed neurons may have topological errors that go unnoticed. One common case is the problem with unconnected neuron segments that generate errors in the analyses.

### Potential solution

A simple solution in neuTube is to select all nodes by pressing **Ctrl+A** and the pressing **C** to connect the fragments. This process may alter the position of the root. If the root is changed, selecting the original root (in the soma) and setting it as root again will solve the problem. Alternatively, if the fragment can be easily identified, the specific pair of nodes can be individually connected.

### Problem 2

**TREES Toolbox not running** (steps 17, 20, and 24)**.**

### Potential solution

Make sure that the ***tre_start.m*** script is correctly pointing to the TREES Toolbox directory.

### Problem 3

**Quantification results are inaccurate for neuron type-specific analysis** (steps 20 and 24)**.**

### Potential solution

Confirm that the multi-signal swc files from different neuron types are not mixed together for a group-wise analysis. Remove the reconstruction files of one neuron type from the analysis folder before placing files from another neuron type.

## Resource availability

### Lead contact

Further information and request of resources should be directed to Giorgio A. Ascoli (ascoli@gmu.edu).

### Materials availability

All unique/stable reagents generated in this study are available from the Cox Lab without restriction. Requests for these reagents can be submitted to Daniel N. Cox (dcox18@gsu.edu).

### Data and code availability

All image stacks, data, analysis, and modeling code have been deposited to Mendeley Data: [https://doi.org/10.17632/wpzd2wxtgn.1], Neuronal reconstructions are available at NeuroMorpho.Org (Ascoli and Cox archives).
